# Supplementary material for: Nectin-1 Expression Correlates with the Susceptibility of Malignant Melanoma to Oncolytic Herpes Simplex Virus In Vitro and In Vivo
Source: Cancers (Basel). 2021 Jun 19;13(12):3058. doi: 10.3390/cancers13123058 (PMC8234279; doi:10.3390/cancers13123058)
Supplement: Supplementary file 1 [file cancers-13-03058-s001.zip › cancers-1257852-supplementary.pdf]

Supplementary Figure 1.

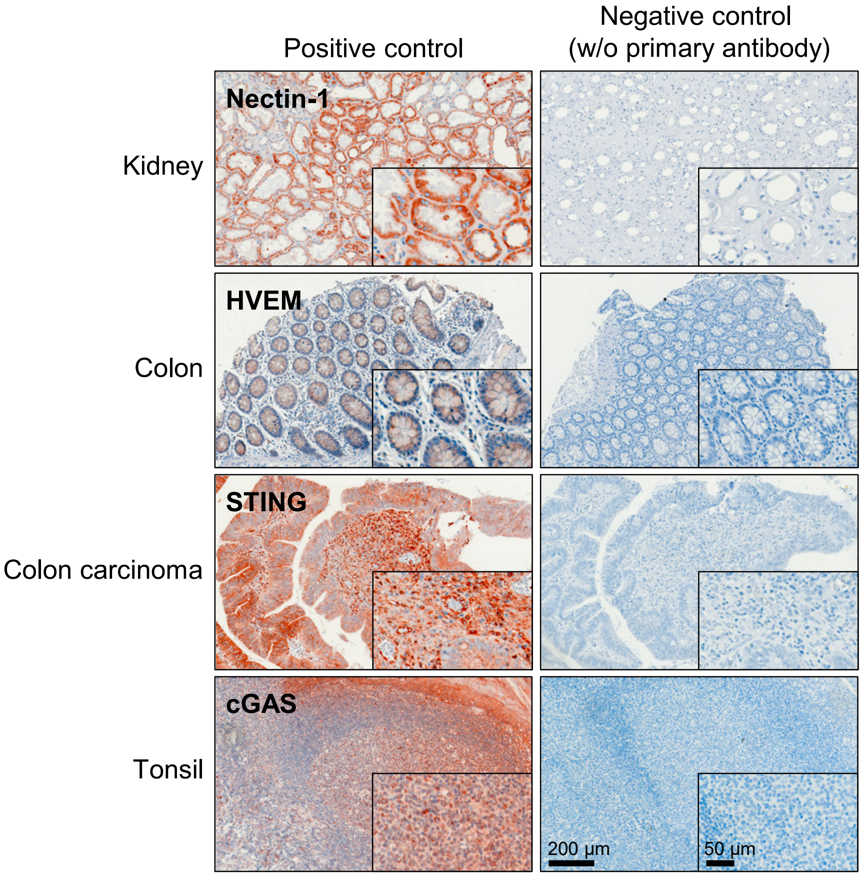

Figure S 1. Optimization of immunohistochemistry staining protocols for Nectin-1, HVEM, STING, and cGAS. Organ slices containing positive control tissues (kidney, colon, colon carcinoma and tonsil) were stained with the respective antibodies and probed with peroxidase-conjugated anti-rabbit or anti-mouse IgG. Negative controls were stained with secondary antibodies only. Images with corresponding size bars show an overview of immunohistochemistry staining and details at higher magnification (insert).

Supplementary Figure 2.

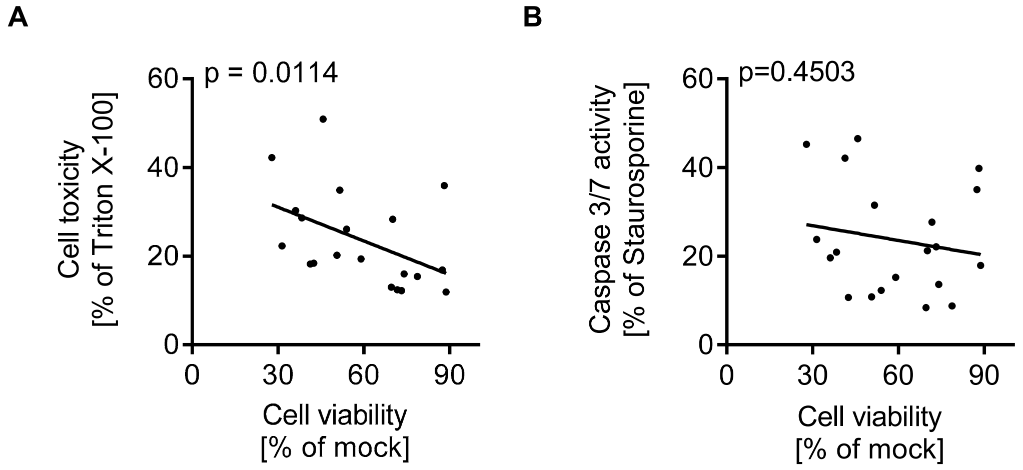

Figure S 2. Correlation analysis of cell viability with toxicity and Caspase 3/7 activity in a panel of 20 melanoma cell lines after T-VEC infection (MOI 1). Cell viability as measured by MTT viability assay was correlated with **(A)** toxicity as evaluated by the release of lactate dehydrogenase (LDH) in infected cells and **(B)** caspase 3/7 activity, as described in the Materials and Methods section. Spearman correlation coefficient analysis was performed comparing the mean of two separate experiments for LDH and caspase 3/7 activity assays with the mean of three separate experiments using MTT viability assay.

Supplementary Figure 3.

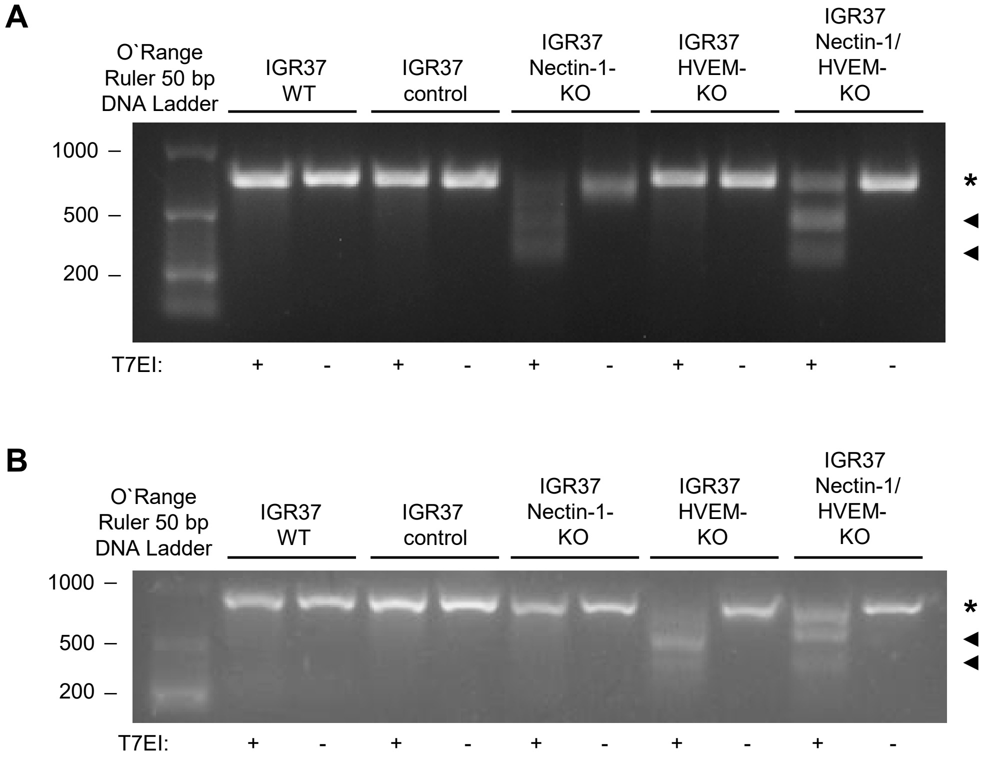

Figure S 3. Evaluation of CRISPR/Cas9 efficiency in different knockout cell lines using T7 endonuclease I (T7EI) assay. Genomic DNA was prepared from the indicated cell lines to amplify the regions flanking the CRISPR sites for either **(A)** Nectin-1 or **(B)** HVEM and tested for CRISPR/Cas9-induced mutations by T7EI digestion where indicated. T7EI cleavage products were analyzed by agarose gel electrophoresis. Amplicons are marked by an asterisk, T7E1 cleavage products by arrowheads.

Supplementary Figure 4.

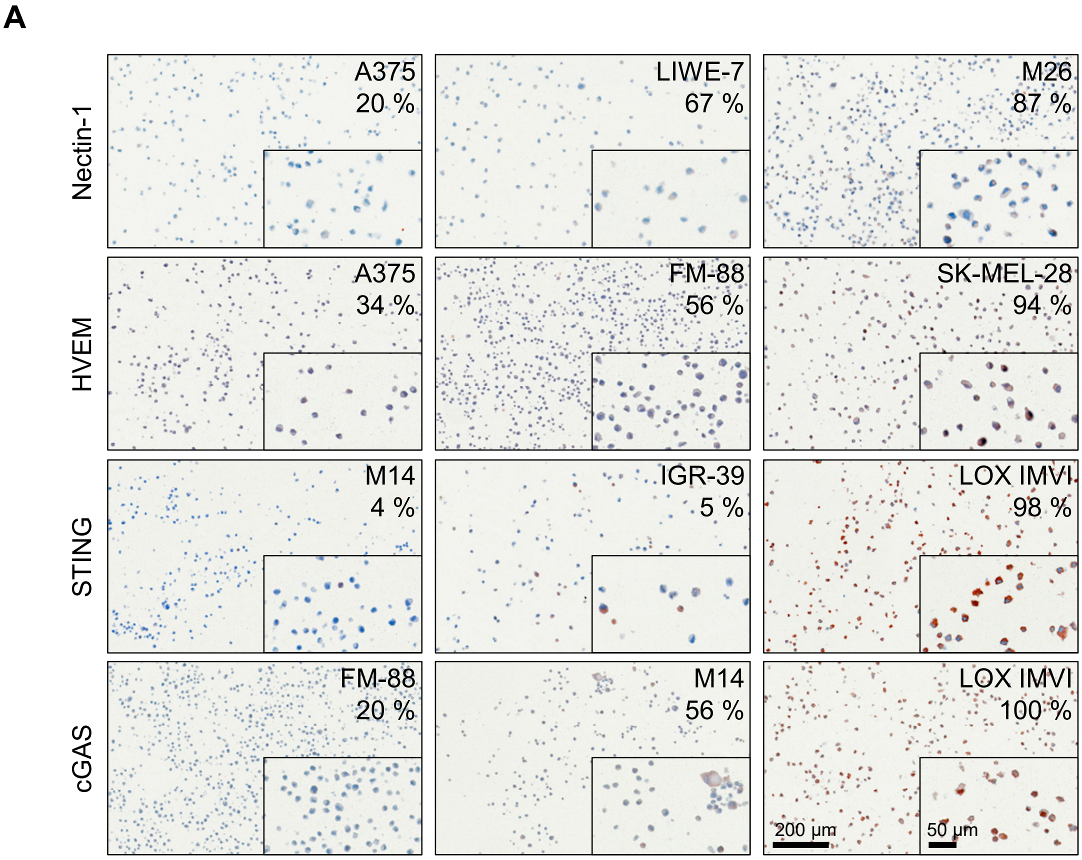

Figure S4. Cont.

B

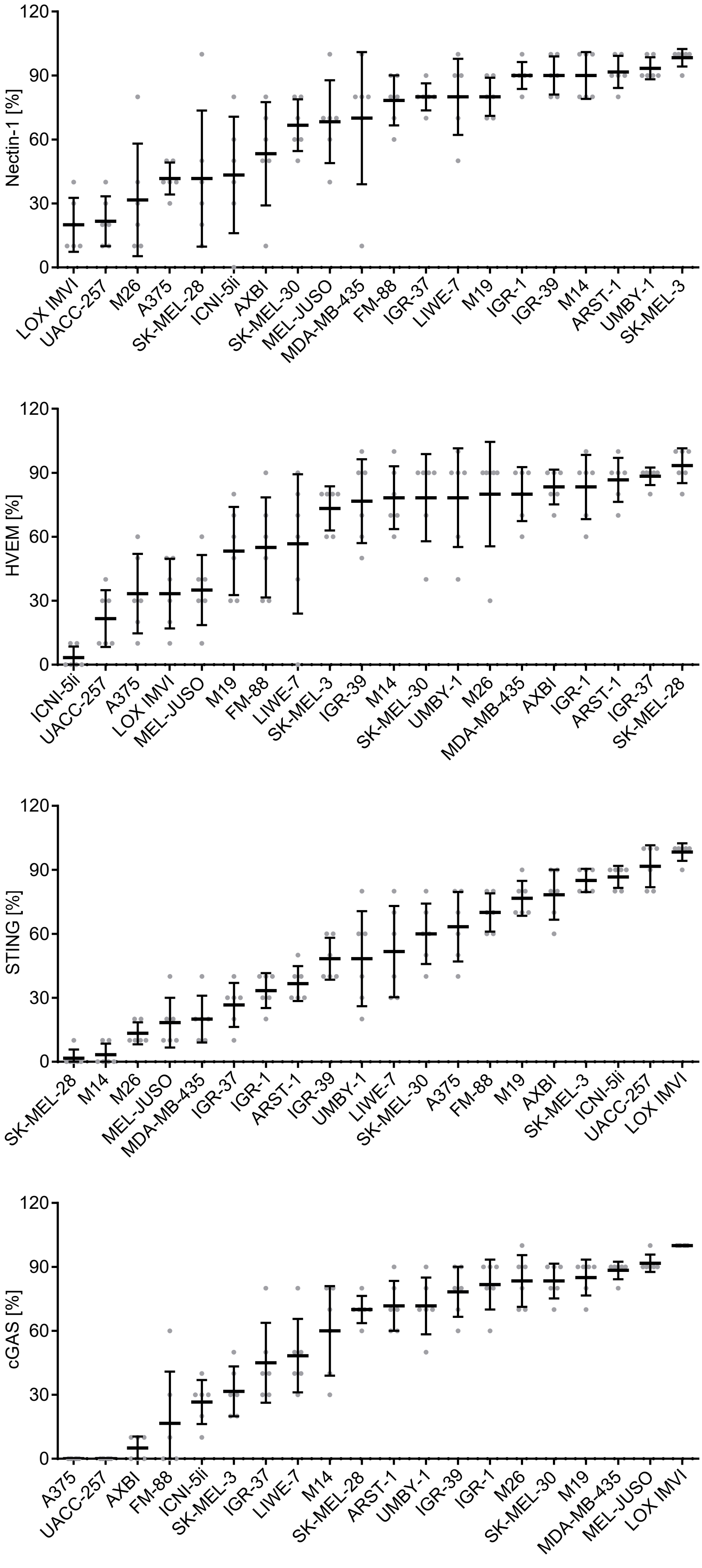
Nectin-1 [%]
0
30
60
90
120
LOX IMVI
UACC-257
M26
A375
SK-MEL-28
ICNI-5ii
AXBI
SK-MEL-30
MEL-JUSO
MDA-MB-435
FM-88
IGR-37
LIWE-7
M19
IGR-1
IGR-39
M14
ARST-1
UMBY-1
SK-MEL-3
HVEM [%]
0
30
60
90
120
ICNI-5ii
UACC-257
A375
LOX IMVI
MEL-JUSO
M19
FM-88
LIWE-7
SK-MEL-3
IGR-39
M14
SK-MEL-30
UMBY-1
M26
MDA-MB-435
AXBI
IGR-1
ARST-1
IGR-37
SK-MEL-28
STING [%]
0
30
60
90
120
SK-MEL-28
M14
M26
MEL-JUSO
MDA-MB-435
IGR-37
IGR-1
ARST-1
IGR-39
UMBY-1
LIWE-7
SK-MEL-30
A375
FM-88
M19
AXBI
SK-MEL-3
ICNI-5ii
UACC-257
LOX IMVI
cGAS [%]
0
30
60
90
120
A375
UACC-257
AXBI
FM-88
ICNI-5ii
SK-MEL-3
IGR-37
LIWE-7
M14
SK-MEL-28
ARST-1
UMBY-1
IGR-39
IGR-1
M26
SK-MEL-30
M19
MDA-MB-435
MEL-JUSO
LOX IMVI

Figure S 4. Evaluation of Nectin-1, HVEM, STING, and cGAS immunohistochemistry in melanoma cell lines. (A) Paraffin-embedded melanoma cell lines were stained with respective antibodies and peroxidase-conjugated anti-rabbit or anti-mouse IgG, as outlined in the Methods section. Images with corresponding size bars show an overview and details at higher magnification (inserts). Six individuals classified the percentage of stained cells into 11 categories (0%, 1-10%, …, 91-100%); the mean value is given. **(B)** Variability of scoring showing mean and standard deviation plus minimum and maximum for each lesion, ranked with ascending positivity for each marker.

Supplementary Figure 5.

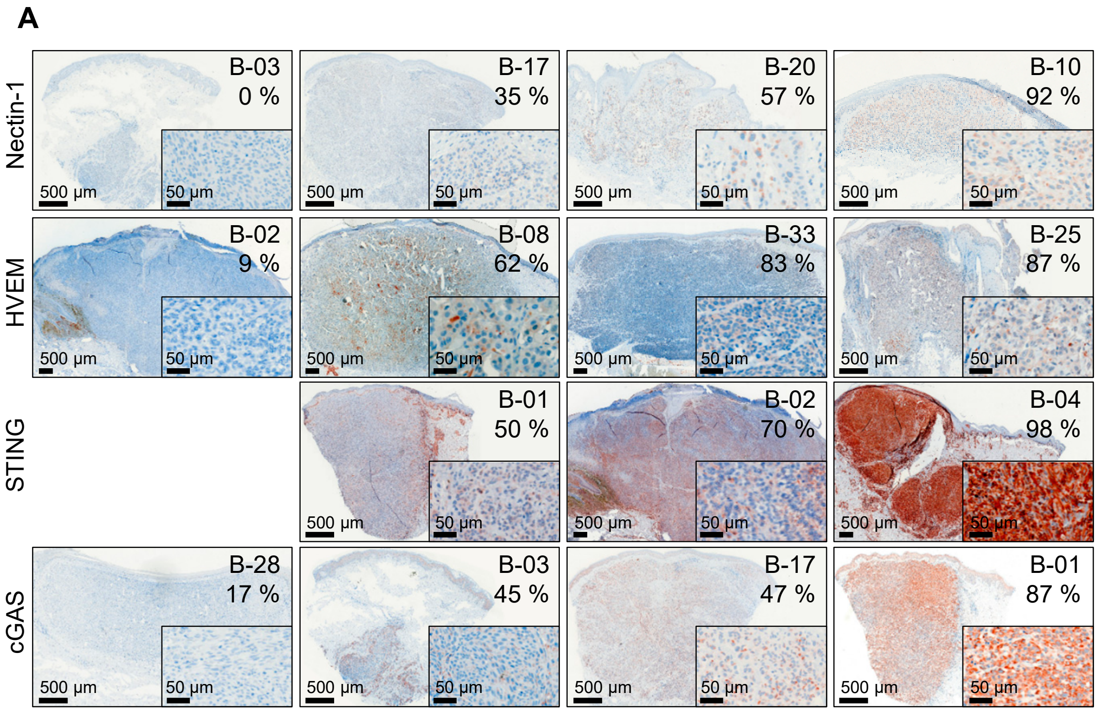

Figure S5. Cont.

B

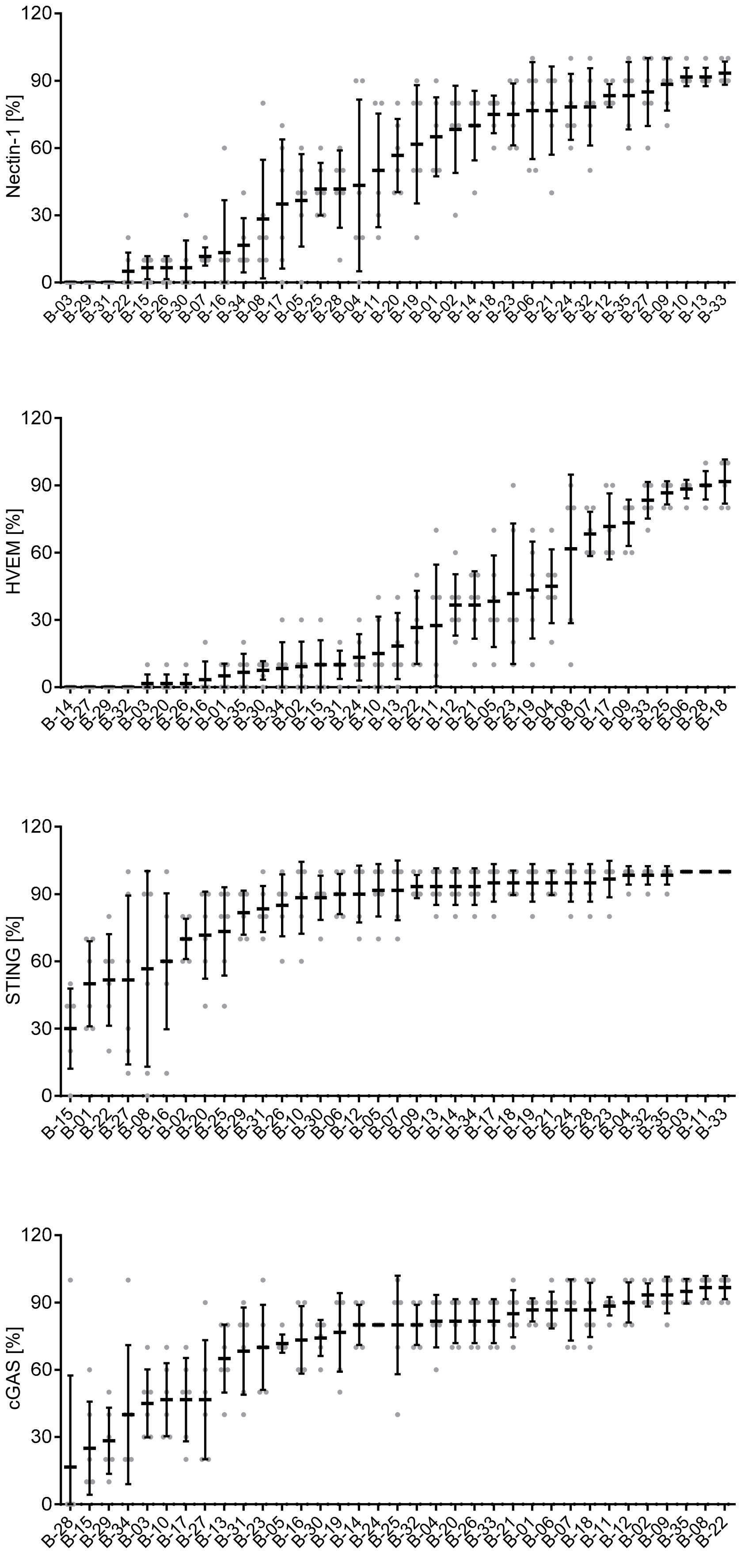
Nectin-1 [%]
120
90
60
30
0
B-03 B-29 B-31 B-22 B-15 B-26 B-30 B-07 B-16 B-34 B-08 B-17 B-05 B-25 B-28 B-04 B-11 B-20 B-19 B-01 B-02 B-14 B-18 B-23 B-06 B-21 B-24 B-32 B-12 B-35 B-27 B-09 B-10 B-13 B-33
HVEM [%]
120
90
60
30
0
B-14 B-27 B-29 B-32 B-03 B-20 B-26 B-16 B-01 B-35 B-30 B-34 B-02 B-15 B-31 B-24 B-10 B-13 B-22 B-11 B-12 B-21 B-05 B-23 B-19 B-04 B-08 B-07 B-17 B-09 B-33 B-25 B-06 B-28 B-18
STING [%]
120
90
60
30
0
B-15 B-01 B-22 B-27 B-08 B-16 B-02 B-20 B-25 B-29 B-31 B-26 B-10 B-30 B-06 B-12 B-05 B-07 B-09 B-13 B-14 B-34 B-17 B-18 B-19 B-21 B-24 B-28 B-23 B-04 B-32 B-35 B-03 B-11 B-33
cGAS [%]
120
90
60
30
0
B-28 B-15 B-29 B-34 B-03 B-10 B-17 B-27 B-13 B-31 B-23 B-05 B-16 B-30 B-19 B-14 B-24 B-25 B-32 B-04 B-20 B-26 B-33 B-21 B-01 B-06 B-07 B-18 B-11 B-12 B-02 B-09 B-35 B-08 B-22

Figure S 5. Evaluation of Nectin-1, HVEM, STING, and cGAS immunohistochemistry in biopsies from melanoma lesions. (A) Range of positivity for the four biomarkers in pretreatment biopsies stained with respective antibodies and peroxidase-conjugated anti-rabbit or anti-mouse IgG, as outlined in the Methods section. Images with corresponding size bars present an overview and details at higher magnification (inserts). **(B)** Evaluation of the immunostaining by six independent individuals, who categorized the percentage of stained cells for each marker (0%, 1-10%, …, 91-100%). Data show mean and standard deviation plus minimum and maximum for each lesion, ranked with ascending positivity for each marker.

Supplementary Figure 6.

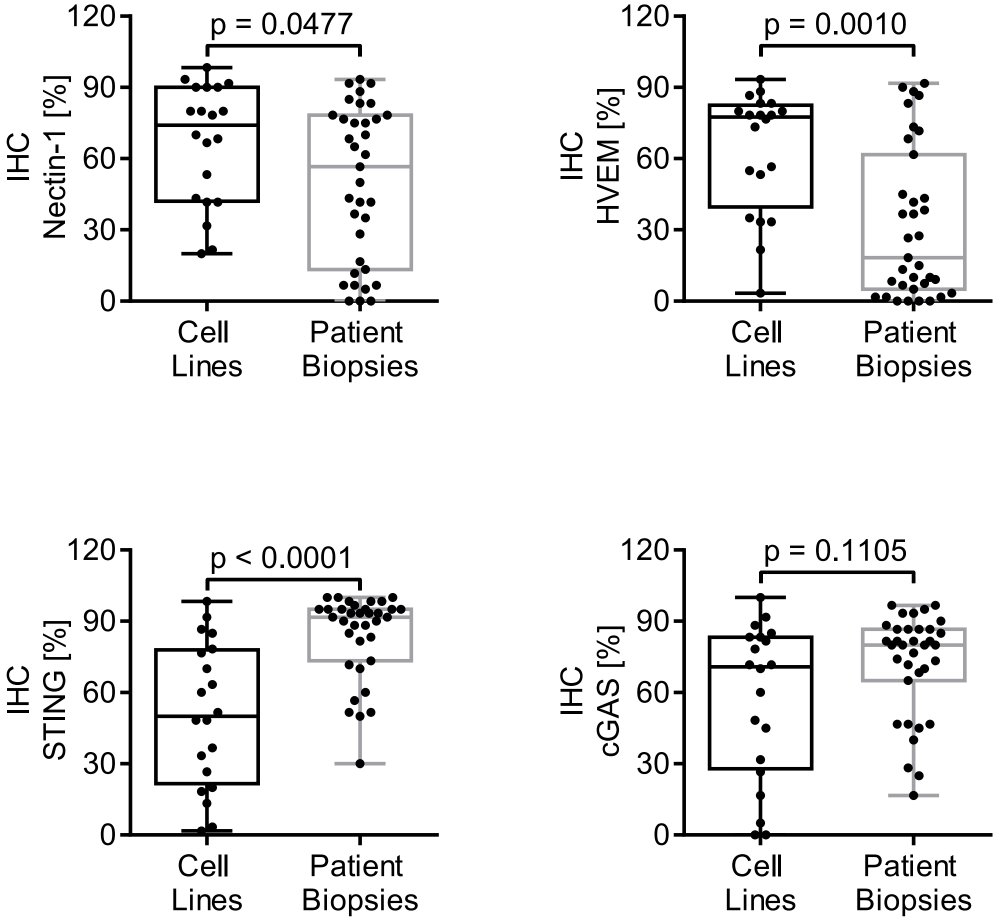

Figure S 6. Comparison of Nectin-1, HVEM, STING, and cGAS expression levels in melanoma cell lines and melanoma metastasis. Range of positivity (%) for the four biomarkers after staining of paraffin-embedded melanoma cell lines and pretreatment biopsies using immunohistochemistry (IHC) as outlined in the Methods section. Box plots show median and interquartile ranges in addition to minimum and maximum values; statistics was calculated using the Mann-Whitney test.

Supplementary Figure 7.

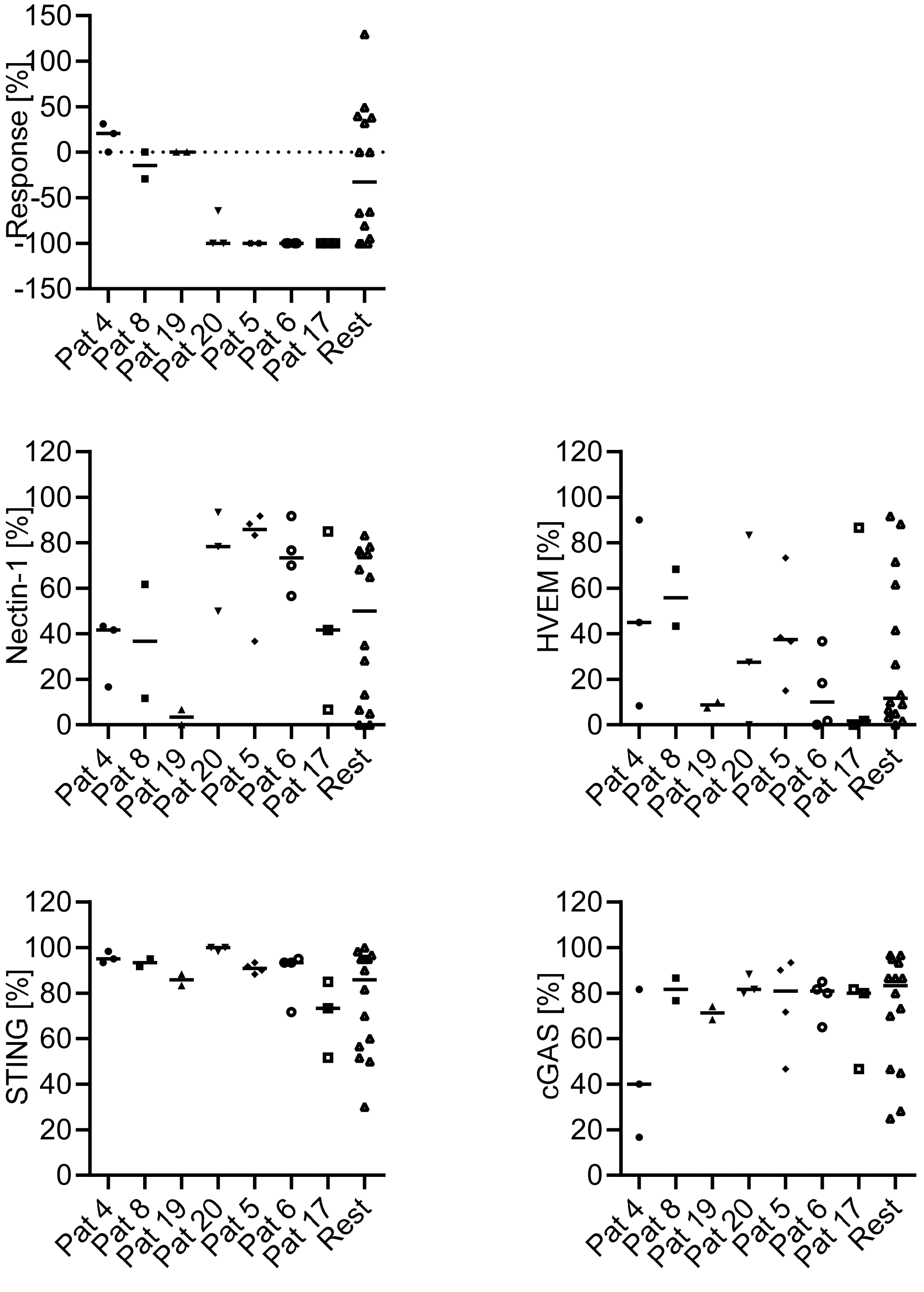

Figure S 7. Clinical response to T-VEC injection and expression levels of biomarkers in patients with two or more biopsies before treatment. The symbols represent metastases of the individual patients, showing the degree of tumor regression upon T-VEC injection and the percentage (%) of cells expressing Nectin-1, HVEM, STING, and cGAS. Horizontal bars represent median values.
